# Energy-Efficient Data Gathering Scheme Based on Broadcast Transmissions in Wireless Sensor Networks

**DOI:** 10.1155/2013/402930

**Published:** 2013-08-24

**Authors:** Soobin Lee, Howon Lee

**Affiliations:** ^1^Institute for IT Convergence, KAIST, Yuseong-gu, Daejeon 305-701, Republic of Korea; ^2^Department of Electrical, Electronic and Control Engineering, Hankyong National University, Anseong, Gyeonggi 456-749, Republic of Korea

## Abstract

Improving energy efficiency is the most important challenge in wireless sensor networks. Because sensing information is correlated in many sensor network applications, some previous works have proposed ideas that reduce the energy consumption of the network by exploiting the spatial correlation between sensed information. In this paper, we propose a distributed data compression framework that exploits the broadcasting characteristic of the wireless medium to improve energy efficiency. We analyze the performance of the proposed framework numerically and compare it with the performance of previous works using simulation. The proposed scheme performs better when the sensing information is correlated.

## 1. Introduction

Energy efficiency is the most important issue in wireless sensor networks because sensor nodes are usually battery-powered, and in many sensor network applications, it is not possible to replenish the energy of sensor nodes. To improve energy efficiency and extend the network lifetime of sensor networks, many proposals have been made for preventing redundant information from being transmitted and received by exploiting the spatial correlation between the sensing information gathered by sensor nodes. Typically, sensor nodes are deployed densely to achieve satisfactory coverage; hence, the sensing information gathered by sensor nodes is highly correlated [1–3].

Depending on the sensor network applications that are used, there exist two approaches to exploiting the correlation between sensing information. The first approach is used when the goal of the sensor network application is to estimate an event from the sensor field with a certain reliability at a sink node [4–6]. In this approach, only some of the nodes transmit their sensed information to the sink node. The second approach is used in which the sensed information at each location is equally important, such as an environmental monitoring application or video surveillance system. This approach typically uses joint coding of correlated information to compress the data. The distributed source coding (DSC) technique [7] allows sensor nodes to use joint coding without explicit communication between the nodes. This technique makes it easy to find the optimal transmission structure. However, the use of DSC in large-scale networks encounters practical problems because this technique requires complex encoders and global knowledge of the network.

In view of these practical difficulties, [8, 9] proposed ideas for compressing correlated sensed information using joint coding with explicit communications between nodes, which is called the explicit communication approach. In [8], it is claimed that there is no need to impose the constraint that the encoding should be performed without sharing information from the other nodes because nodes receive data from the other nodes in each data gathering path to the sink node. Reference [9] addressed an optimization problem for the minimum cost correlated data gathering tree (MCCDGT) and proposed distributed heuristic approximation algorithms to solve this problem.

In the previous works that use the explicit communication approach, a communication channel is abstracted as a point-to-point link, which ignores the fact that wireless channels transmit information by broadcasting it, which makes it available to any receiver of the right type. In wireless networks, when one node transmits data to its destination node, other nodes within the transmission range of the transmitting node can also receive the data and may use it to compress their own information. Herein, we propose a framework for data compression that exploits the broadcasting characteristic of the wireless medium, thereby achieving greater energy efficiency.

The remainder of this paper is organized as follows. In Section 2, we review related work. In Section 3, we explain the proposed scheme and analyze its performance numerically. In Section 4, we evaluate the performance of the proposed scheme using simulations. In Section 5, we consider MAC protocols for the proposed scheme.  Section 6 concludes.

## 2. Related Work

References [4–6] exploit the broadcasting characteristic of the wireless medium to reduce the energy consumption. In these works, if one node transmits its sensing information, the other nodes within transmission range overhear the information. They then determine whether or not their own information is redundant, either by (i) checking that the distance from the transmitting node is less than the correlation radius [4] or the influential range [5] or by (ii) spatial interpolation using the data that they overheard [6]. The distance between sensor nodes is assumed to be known from the exchange of messages during network initialization or estimated using the received signal strength. If a node determines that its sensing information is redundant, it does not try to transmit the information. Consequently, only some of the total nodes transmit their sensing information to the sink node, while satisfying a certain distortion constraint; hence, the amount of energy that is consumed by transmitting redundant information is reduced. However, the schemes that are proposed in these works cannot be used for the wireless sensor network applications in which all information that is collected from the sensor field is equally important and should be sent to the user of the application, such as environmental monitoring systems or video surveillance systems.

In [9], the authors considered a sensor network as a connectivity graph with point-to-point communication links instead of a full wireless multipoint communication structure. They formulated the optimization problem for minimizing the network's energy consumption by jointly optimizing the data gathering structure and data compression. This problem is called the MCCDGT problem. Due to the fact that this optimization problem is NP-hard, the authors proposed a number of distributed heuristic approximation algorithms for solving it.

Herein, we compare the performance of our proposed scheme to the performance of MCCDGT whereas in [9], nodes compress their information only using data that they have received from their child nodes in the data gathering structure; in the proposed scheme, nodes can also compress their information using the data that they have received from neighbor nodes that are not their child nodes. We separate the data gathering structure and the data compression scheme to fully exploit the broadcasting characteristic of the wireless medium. Furthermore, we use a widely used spatial correlation model to consider more realistic environments though a simplified version of the correlation model is used in [9].

## 3. Energy-Efficient Data Gathering Based on Broadcast Transmissions

### 3.1. Assumptions

We consider sensor network applications in which sensing information at each sensor node is equally important; hence, all sensor nodes measure the environmental variables and transmit the sensing information to the sink node periodically. In addition, we assume that *N* sensor nodes are deployed randomly according to a Poisson point process.

#### 3.1.1. Model of Energy Behavior of Nodes

A simple model of the energy behavior of sensor nodes is introduced in [10]. This model assumes a path loss of (1/*d*)^*n*^, where *d* is the distance between a sender and a receiver, and uses the following definitions: *α*_11_ is the energy per bit consumed by the transmitter electronics, *α*_2_ is the energy per bit per *m* ^*n*^ used in the transmitter amplifier, *α*_12_ is the energy per bit consumed by the receiver electronics, and *α*_3_ is the energy used to sense a bit. Then, the energy consumed by a transmitter and a receiver that are separated by a distance of *d* to transport one bit is represented as

(1) $$\begin{array}{c} P_{\text{relay}}\left(d\right)=\alpha_{11}+\alpha_{2}d^{n}+\alpha_{12}=\alpha_{1}+\alpha_{2}d^{n}, \end{array}$$

where *α*_1_ = *α*_11_ + *α*_12_. Using this model as a basis, the author demonstrates that the energy required to relay a bit over the distance *L* is bounded as

(2) $$\begin{array}{c} E\left(L\right)\geq \alpha_{1}\frac{n}{n-1}\frac{L}{d_{m}}-\alpha_{12} \end{array}$$

with equality if and only if *L* is an integral multiple of *d*_*m*_, where *d*_*m*_ is the optimal distance between intervening nodes, which is given by

(3) $$\begin{array}{c} d_{m}=\sqrt[{n}]{\frac{\alpha_{1}}{\alpha_{2}\left(n-1\right)}}. \end{array}$$

#### 3.1.2. Data Compression Model

There are several correlation models that describe the spatial correlation of sensed information or methods that estimate the correlation in the wireless sensor networks [1–3]. Herein, we use the power exponential model for simplicity. The model is represented as

(4) $$\begin{array}{c} K_{ji}=e^{-\gamma d_{ji}^{\theta }},\;\theta \in \left\{1,2\right\}, \end{array}$$

where *K*_*ji*_ and *d*_*ji*_ are the correlation coefficient and the distance between node *j* and node *i*, respectively. *γ* indicates the degree of spatial correlation; its value depends on the sensor network application that is used.

To evaluate the amount of compressed data, we consider the relation between spatial correlation and data compression. Let random variables *X*_1_, *X*_2_,…, *X*_*N*_ be the sensing information from each sensor node. As in [9], *X*_1_, *X*_2_,…, *X*_*N*_ are entropy coded with *h*_*X*_ = *h*(*X*_1_) = *h*(*X*_2_)⋯ = *h*(*X*_*N*_) if there is no explicit information from other nodes. For the maximum possible lossless compression, each node's information is compressed by conditional entropy coding, given the data that is received from other nodes [9, 11]. Given our assumption that the conditional entropy is characterized by the correlation model in (4), the conditional entropy *h*(*X*_*j*_ | *X*_*i*_) is given by

(5) $$\begin{array}{c} h\left(X_{j}\;|\;X_{i}\right)\left(d_{ji}\right)=\left(1-K_{ji}\right)h_{X}=\left(1-e^{-\gamma d_{ji}^{\theta }}\right)h_{X}, \end{array}$$

where 1 − *e* ^−*γd*_*ji*_^*θ*^^ is the compression ratio. After it has received the compressed data, the sink node can decode it if the information for identifying the source, which is used in joint coding as the explicit information, is included.

### 3.2. Proposed Data Gathering Scheme

We propose a distributed data compression scheme based on wireless point-to-multipoint communication. We use the restriction that each sensor node is allowed to compress its own sensing information, using only the data that is not compressed. In addition, we assume that the data gathering structure is already constructed using the optimal algorithm. In the proposed scheme, raw data transmitter (RDT) nodes transmit their sensing information without compression. Nodes that are not chosen as RDT nodes compress their information using only the data received from the RDT nodes.

The proposed scheme has two phases. In the first phase, which is called the *RDT selection phase*, RDT nodes are chosen among *N* sensor nodes. Each sensor node becomes a RDT node with probability *β*, where 0 ≤ *β* ≤ 1. After the RDT nodes are selected, each non-RDT node selects its RDT node. RDT nodes broadcast advertising messages to their neighbor nodes. Non-RDT nodes that receive those advertising messages estimate the distance to the RDT nodes on the basis of the received signal strength of the advertising messages. Let *S* be the set of all sensor nodes and *S*_*R*_ the set of nodes that are chosen as RDT nodes. Then, the set of non-RDT nodes is given by *S*_NR_ = *S*∖*S*_*R*_. Let *C*_*j*_ be the set of RDT nodes from which non-RDT node *j* ∈ *S*_NR_ receives advertising messages. Node *j* chooses its RDT node *r*_*j*_ that satisfies *r*_*j*_ = arg min⁡_*i*∈*R*_*j*__ *d*_*ji*_ from the nodes in

(6) $$\begin{array}{c} R_{j}=\left\{i\in C_{j}\;|\;d_{ji}\leq R_{\text{th}}\right\}, \end{array}$$

where *d*_*ji*_ is the estimated distance between node *j* and node *i* and *R*_th_ is the predefined threshold distance. Because the correlation between sensing information that is generated at two sensor nodes increases when the distance between those two nodes decreases, sensing information is mostly compressed using the data from the nearest RDT node, which minimizes the energy consumed to send the compressed data to the sink node.

In the second phase, which is called the *data gathering phase*, all sensor nodes send their sensing information to the sink node through the data gathering structure. As mentioned above, RDT nodes transmit their sensing information without compression. However, not all the data that the RDT nodes transmit is sensing information generated at the RDT nodes because sensor nodes also relay the data that is received from child nodes in their data gathering paths. Non-RDT nodes need to distinguish the data that is generated at their RDT nodes from the data that is just relayed by the RDT nodes. To do this, additional information that indicates whether or not the corresponding sensing information is generated at the transmitting node is required; for example, we can add a new field to RTS frames in CSMA-based MAC protocols, such as IEEE 802.11 or S-MAC [12]. After receiving a RTS frame, neighbor nodes can detect whether the information in received data frames following the RTS frame is generated at the transmitting node or not.

Further, because non-RDT nodes should transmit their own information after receiving data from their RDT nodes and compressing the information using the received data, the transmission of data that is generated at RDT nodes should be given priority over the transmission of data that is generated at non-RDT nodes and has not yet been compressed. For example, in TDMA-based MAC protocols, the priority scheme can be set up by scheduling the transmissions of RDT nodes first. In CSMA-based MAC protocols, we can use a priority-based transmission policy as follows. RDT nodes or non-RDT nodes that do not have RDT nodes participate in contention to acquire a channel when they have data to transmit. Non-RDT nodes that have RDT nodes participate in contention only when they have either (i) data that is generated by them and that they have already compressed using the data received from their RDT nodes or (ii) data that needs to be relayed.

**Figure 1:**
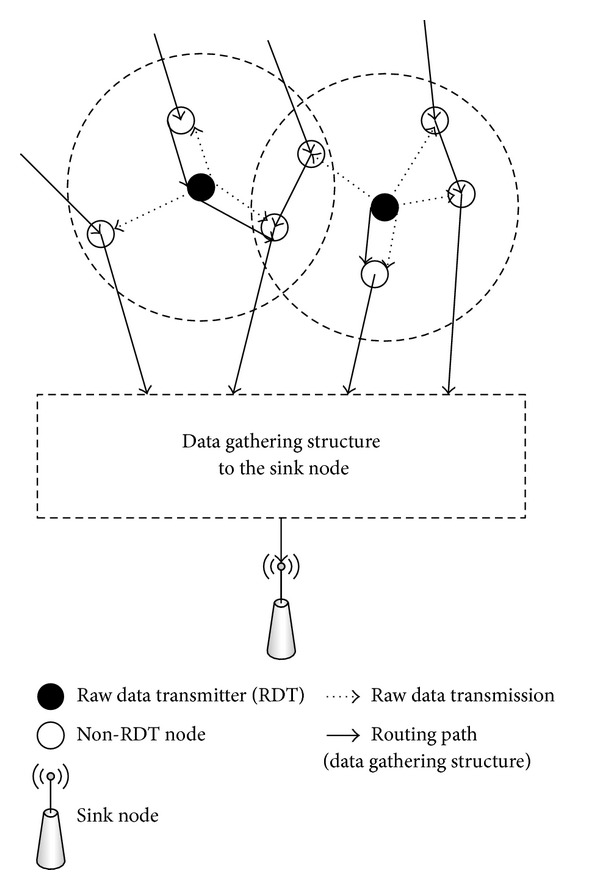
Proposed data compression framework.

Let *P*(*k*) be a parent node of node *k* ∈ *S* in the data gathering structure. During the data gathering phase, node *m* ∈ *S* always receives the data from node *k* ∈ *S* if *m* = *P*(*k*). For nodes *j* ∈ *S*_NR_ and *j* ≠ *P*(*r*_*j*_), when *j* detects *r*_*j*_'s transmission, *j* checks whether or not *r*_*j*_ has generated its data itself. If *r*_*j*_'s data is locally generated, *j* receives it. If not, *j* turns off the radio and enters the idle state until the current transmission is complete. After receiving *r*_*j*_'s data, *j* compresses its own information using joint coding, given the *r*_*j*_ data. If *j* does not have *r*_*j*_ or it does not receive the data from *r*_*j*_ correctly within the prespecified duration, *j* transmits its sensing information without compression. The proposed framework is illustrated in Figure 1.

Several different definitions are in use for the network lifetime; for example, the interval from the time at which the sensor network starts its operation to the time at which the first sensor dies, when the number of active nodes falls below a prespecified threshold, or when the sensing coverage falls below a prespecified threshold [13, 14]. In general, the definition of network lifetime that is appropriate to use in any given situation may depend on the wireless sensor network application in question. However, whatever definition is used, balancing the energy usage across sensor nodes extends the network lifetime. In the proposed scheme, RDT nodes consume more energy than non-RDT nodes because they transmit uncompressed data. To prevent some sensor nodes from dying much earlier than the rest of nodes and to extend the network lifetime, RDT nodes can be selected periodically. Reference [9] does not attempt to balance the energy of sensor nodes.

### 3.3. Numerical Analysis

Let *D* be the deployment area of the sensor network. Each non-RDT node chooses its RDT node from the RDT nodes that lie within *R*_th_. Thus, the probability of a non-RDT node in *D* being covered by its RDT node, *P*_NR,cov⁡_, can be given by

(7) $$\begin{array}{c} P_{\text{NR},\mathrm{cov}}=1-e^{-(\beta N/D)\pi R_{\text{th}}^{2}} \end{array}$$

using a theorem in [15, 16]. Then, the number of non-RDT nodes that are covered by the RDT nodes is

(8) $$\begin{array}{c} N_{\text{NR}}=\left(1-\beta \right)NP_{\text{NR},\mathrm{cov}}. \end{array}$$

Let a random variable *R*_min⁡_ be the distance between any arbitrary non-RDT node and the nearest RDT node. The distribution density function of *R*_min⁡_ is given by

(9) $$\begin{array}{c} f_{R_{\min}}\left(d\right)=\frac{\beta N}{D}2\pi de^{-(\beta N/D)\pi d^{2}} \end{array}$$

as in [15]. Then, the average number of bits to be transmitted by each non-RDT node that is covered by its RDT node after compression is

(10) havg,NR=∫0RtfRmin⁡(x)h(Xj ∣ Xi)(x)dx=2πβNhXD∫0Rtxe−(βN/D)πx2(1−e−γxθ)dx.

If *θ* = 2, that is, if the correlation model is squared exponential, *h*_avg,NR_ is given by

(11) havg,NR=πβNhX×(1−e−(πβN/D)Rth2πβN+−1+e−(πβN/D+γ)Rth2πβN+γD).

On the other hand, RDT nodes or non-RDT nodes that are not covered by RDT nodes transmit their sensing information without compression. Consequently, we can obtain the average number of bits to be transmitted by each node in the network as follows:

(12) $$\begin{array}{c} h_{\text{avg}}=\frac{N_{\text{NR}}h_{\text{avg},\text{NR}}+\left(N-N_{\text{NR}}\right)h_{X}}{N}. \end{array}$$

Now, we calculate the total energy consumed by sensor nodes to transport all the information possessed by one sensing event to the sink node, *E*_total_. Given that *E*(*L*) in (2) is the minimum energy required to forward a bit over the distance *L*, *E*_total_ can be represented as

(13) $$\begin{array}{c} E_{\text{total}}=N\alpha_{3}h_{X}+\frac{N}{D}\iint_{D}E\left(d\right)h_{\text{avg}}dS+N_{\text{NR}}\alpha_{12}h_{X} \end{array}$$

if we use an ideal data gathering structure. The first term on the right-hand side covers the energy consumed by sensor nodes to sense an event and the third term covers the energy consumed by non-RDT nodes to receive data from their RDT nodes. For simplicity, we assume that the deployment area is disk-shaped and that the sink node is located in the center of the deployment area. If we let *r*_*D*_ be the radius of *D*, we have

(14) Etotal=(Nα3+NNRα12)hX+Nhavg(α1n(n−1)dm23rD−α12).

## 4. Performance Evaluation

We now verify the numerical analysis by simulation and compare the performance of the proposed scheme with that of MCCDGT. The authors in [9] used a simplified model for the data correlation, in which the compression ratio is constant. Herein, we use the spatial correlation model described in (4), in which the compression ratio varies depending on the distance between nodes; thus, more practical environments are considered. In addition, the weight of each path between sensor nodes is calculated using (1). We set the simulation parameters as follows: *N* = 100, *r*_*D*_ = 30 m, *h*_*X*_ = 10 kbytes, *α*_11_ = 20 nJ/bit, *α*_12_ = 10 nJ/bit, *α*_2_ = 500 pJ/bit/m^2^, and *n* = 2. *α*_3_ is assumed to be negligibly small. The maximum transmission range of sensor nodes is 20 m and *R*_th_ is 10 m.

**Figure 2:**
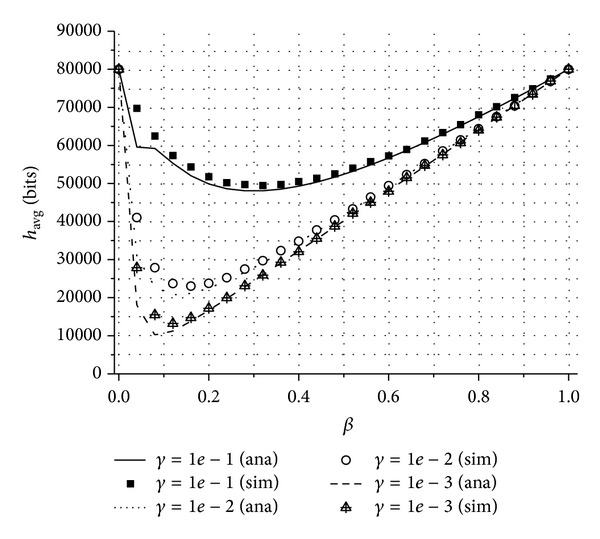
The average number of bits to be transmitted by each node in the network (*h*_avg_) for different *β* and *γ* values.

In Figure 2, we show the average number of bits to be transmitted by each node (*h*_avg_) in the numerical analysis and the simulation results of the proposed scheme for different values of *β* and *γ*. For any given *β*, *h*_avg_ decreases when *γ* is low, that is, when the sensing information of sensor nodes is highly correlated, because the compression ratio of data also becomes lower. On the other hand, for any given *γ*, there exists *β* that minimizes *h*_avg_. If *β* is too small, the number of non-RDT nodes that are covered by their RDT nodes decreases. This means that most of the sensing information cannot be compressed. As a result, *h*_avg_ increases. If *β* exceeds a certain threshold, the number of RDT nodes increases. Consequently, *h*_avg_ also increases because the amount of energy consumed by RDT nodes to transmit uncompressed data becomes larger than the amount of energy saved by compressing data of non-RDT nodes. The numerical results approximate well to those of the simulation.

**Figure 3:**
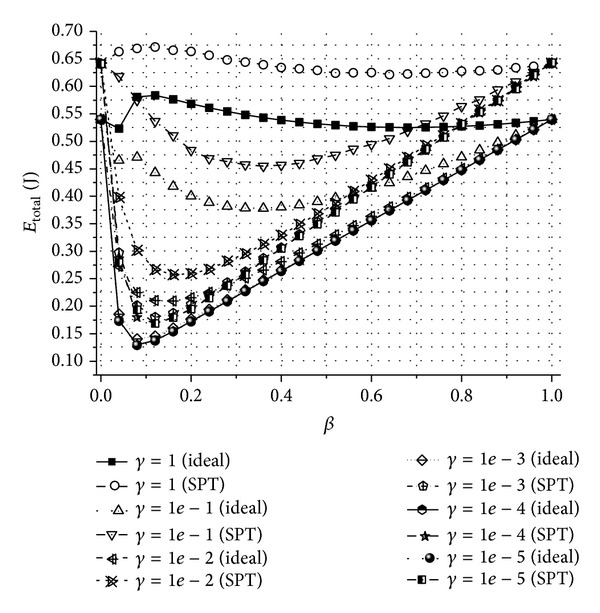
The total energy consumed by sensor nodes to transport all the information derived from one sensing event to the sink node (*E*_total_) for different *β* and *γ* values when an ideal routing and a SPT routing are used.

In Figure 3, we show the total energy consumed by sensor nodes to transport all the information derived from one sensing event to the sink node (*E*_total_) in the proposed scheme, when the ideal data gathering structure and the shortest path tree (SPT) data gathering structure are used. Because the ideal data gathering structure is not available in practice, *E*_total_ in the ideal structure is calculated using (14). In the simulation, we use the SPT structure as the data gathering structure of the proposed scheme. For any given *β* and *γ*, *E*_total_ in the SPT structure is always greater than the ideal case. *E*_total_ in the ideal structure can be considered as the upper bound of *E*_total_ for the proposed scheme. The effects of *β* and *γ* can be explained as in the case of *h*_avg_.

**Figure 4:**
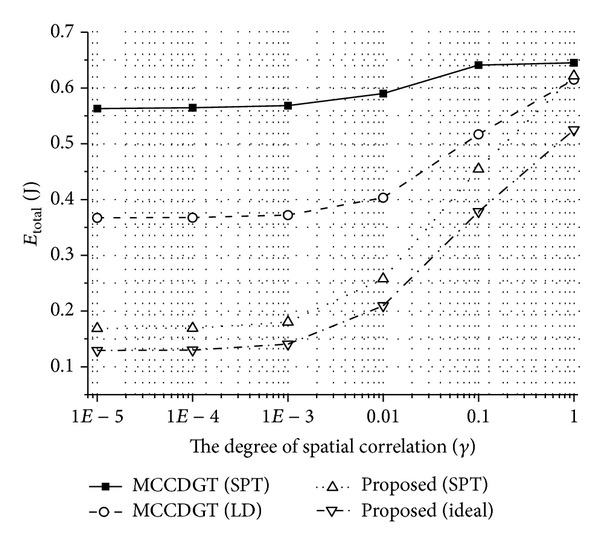
The total energy consumed by sensor nodes to transport all the information derived from one sensing event to the sink node (*E*_total_) for different *γ* values.

In Figure 4, we compare (a) *E*_total_ of the proposed scheme using the ideal and SPT data gathering structures when *β* that minimizes *E*_total_ is used to (b) *E*_total_ of MCCDGT using the leaves deletion (LD) algorithm, which is a fully distributed and practical method. We can find the value of *β* that minimizes *E*_total_ of the proposed scheme using (12). In addition, we show the performance of MCCDGT when using only the SPT algorithm, which is the optimal solution when stores of sensed information are independent of each other. As we can see in Figure 4, the proposed scheme outperforms MCCDGT with the LD algorithm when the sensing information is highly or moderately correlated. If *γ* becomes larger, the performance of the proposed scheme and MCCDGT with the LD algorithm converges to those of MCCDGT with the SPT algorithm because the amount of energy that can be saved by compressing data becomes negligible.

**Figure 5:**
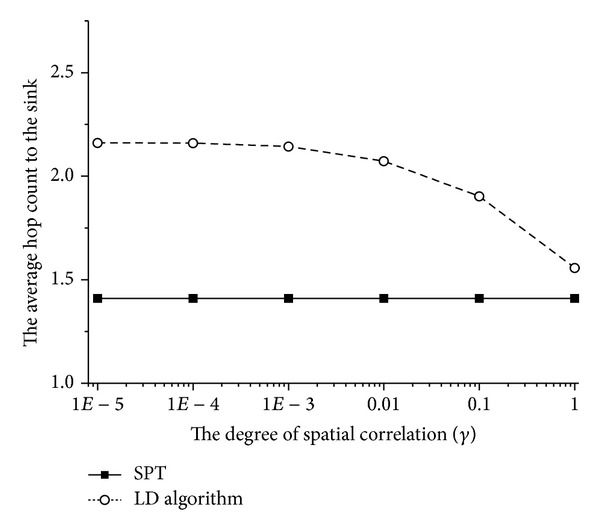
The average hop count from each sensor node to the sink node for different *γ* values.

Due to the fact that we separate the data gathering structure and the data compression scheme, we obtain an additional advantage from the proposed scheme: the average hop count from each sensor node to the sink node can be reduced. In Figure 5, we compare the average hop count to the sink node of the SPT data gathering structure and the structure that is constructed by the LD algorithm. If sensing information is more correlated, more leaf nodes become child nodes of other leaf nodes when the LD algorithm is used because deleting leaf nodes is more energy efficient in that case. Thus, data gathering paths from each sensor node to the sink node are detoured. Consequently, the average hop count to the sink node increases. Meanwhile, we can use an optimal data gathering structure, such as SPT, in the proposed scheme without considering data compression. When the network increases in size, the difference between the average hop count and the sink node in the two structures may be significant. Therefore, the proposed method has an important strength in multimedia sensor network applications that are usually delay sensitive because an increase in the hop count usually causes more delay.

## 5. Consideration for MAC

Many TDMA-based protocols for providing unicast or broadcast transmissions in sensor networks have been proposed. However, TDMA-based protocols have some disadvantages, especially in large-scale multihop networks. They require time synchronization for the entire network and incur overhead for slot allocation. In addition, the schedule needs to be changed when the network topology changes. TDMA-based protocols are suitable for use only in small, specific networks.

In CSMA-based protocols without specific broadcast transmission support, such as IEEE 802.11 or S-MAC [12], the hidden node problem may be significant, depending on the carrier sense threshold [17]. If a collision occurs due to hidden nodes when a non-RDT node is receiving data from its RDT node, the non-RDT node cannot compress its information because the received data is corrupted. As a result, the energy consumed by the non-RDT node to receive the data from its RDT node becomes a loss, and the performance of the proposed scheme decreases, though it still runs normally.

To avoid the degradation due to the hidden node problem, we can tune the sensing threshold of the carrier [17]. However, such tuning may not be possible due to hardware limits; besides, it may delay the transmission of data by nodes. Meanwhile, we can use MAC protocols that support reliable broadcast transmissions, some of which are addressed in [18, 19]. In particular, Robcast [19] guarantees high reliability of broadcast transmissions while keeping low energy consumption regardless of the additional control signaling. We can adapt these protocols to the proposed framework with a few modifications.

## 6. Conclusion

In this paper, we propose a data compression scheme for the wireless sensor networks that exploits the broadcasting characteristic of the wireless medium. We analyze the performance of the proposed scheme numerically and verify the results using simulations. Further, we compare the performance of the proposed scheme with that of MCCDGT. The simulation results show that our scheme outperforms the other schemes when sensing information is correlated. Finally, we discuss broadcast transmission support in the MAC layer for the proposed scheme.
